# The Optimal Blood Pressure Target in Different Dialysis Populations

**DOI:** 10.1038/s41598-018-32281-w

**Published:** 2018-09-20

**Authors:** Jong Hyun Jhee, Jimin Park, Hyoungnae Kim, Youn Kyung Kee, Jung Tak Park, Seung Hyeok Han, Chul Woo Yang, Nam-Ho Kim, Yon Su Kim, Shin-Wook Kang, Yong-Lim Kim, Tae-Hyun Yoo

**Affiliations:** 1 Division of Nephrology and Hypertension, Department of Internal Medicine, Inha University College of Medicine, Incheon, Korea; 2 Department of Internal Medicine, College of Medicine, Institute of Kidney Disease Research, Yonsei University, Seoul, Korea; 3 Department of Internal Medicine, College of Medicine, Severance Biomedical Science Institute, Brain Korea 21 PLUS, Institute of Kidney Disease Research, Yonsei University, Seoul, Korea; 4 Department of Internal Medicine, The Catholic University of Korea College of Medicine, Seoul, Korea; 5 Department of Internal Medicine, Chonnam National University Medical School, Gwangju, Korea; 6 Department of Internal Medicine, Seoul National University College of Medicine, Seoul, Korea; 7 Department of Internal Medicine, Kyungpook National University School of Medicine, Daegu, Korea

## Abstract

Hypertension is common and contributes to adverse outcomes in patients undergoing dialysis. However, the proper blood pressure (BP) target remains controversial and several factors make this difficult. This study aimed to investigate the adequate BP target in patients undergoing prevalent dialysis. Data were retrieved from the Clinical Research Center for End-Stage Renal Disease (2009–2014). 2,299 patients undergoing dialysis were evaluated. Patients were assigned into eight groups according to predialysis systolic blood pressure (SBP). The primary outcome was all-cause mortality. During the median follow-up of 4.5 years, a U-shape relation between SBP and mortality was found. The risk of mortality was increased in the SBP <110 and ≥170 mmHg groups. In subgroup analysis, the risk of mortality was similarly shown U-shape with SBP in subjects with no comorbidities, and no use of antihypertensive agents. However, only lowest SBP was a risk factor for mortality in patients with older, having diabetes or coronary artery disease, whereas highest SBP was an only risk factor in younger patients. In respect of dialysis characteristics, patients undergoing hemodialysis showed U-shape between SBP and mortality, while patients undergoing peritoneal dialysis did not. Among hemodialysis patients, patients with shorter dialysis vintage and less interdialytic weight gain showed U-shape association between SBP and mortality. This study showed that the lowest or highest SBP group had higher risk of mortality. Nevertheless, the optimal target BP should be applied according to individual condition of each patient.

## Introduction

Hypertension is a common clinical problem and contributes to a high risk for adverse clinical outcomes in patients undergoing dialysis^1^. It is well known that strict blood pressure (BP) control could reduce major cardiovascular events and all-cause mortality in the general population^2,3^. Recently, the SPRINT research group also reported that intensive treatment of BP is significantly associated with lower rates of major cardiovascular events and all-cause mortality^4^. However, the pathophysiology and management of hypertension is slightly different in patients undergoing dialysis. In patients on dialysis, most of the previous observational studies reported a U-shaped or even an L-shaped association between BP and all-cause mortality, indicating higher mortality at low BP^5–7^. Thus, the proper BP target for patients undergoing dialysis is still controversial.

Managing BP in patients undergoing dialysis may be hindered by multiple factors, such as age, comorbidities including diabetes mellitus (DM) or coronary artery disease (CAD), use of antihypertensive agents (AHAs), and dialysis characters [dialysis type, dialysis vintage, and poor dry weight (DW) control]^1,8^. In the general population, factors such as aging, DM, or cardiovascular complications may increase arterial stiffness and BP^9–11^. Consequently, strict BP control is recommended to overcome adverse clinical outcomes. However, for patients undergoing dialysis, several studies showed that lower BP is more related to adverse outcomes, especially in those with old age, DM, or cardiovascular complications^6,8^. The use of AHA is one of the BP control strategies even in patients on dialysis. However, some studies reported that use of AHAs can lead to several adverse effects, such as intradialytic hypotension^12,13^. An unexpected point in the use of AHAs among patients on hemodialysis (HD) is that a greater use of antihypertensive medications is paradoxically associated with a higher BP^14,15^. It is likely that excessive medications may limit the opportunity to achieve adequate DW and lead to resistant hypertension through an expanded blood volume. Subsequently, volume overloads induce cardiac stress and increase the risk for adverse cardiovascular outcomes^16,17^. Furthermore, HD and peritoneal dialysis (PD) have different levels of hemodynamics affecting BP^18,19^. In HD, dialysis characters such as dialysis vintage and interdialytic weight gain (IDWG) can also have different effects on cardiovascular outcomes.

Given that BP is one of the major factors for determining prognosis in patients on dialysis, it is important to ascertain the proper BP target and the factors interfering with the management of BP in these patients. Therefore, the objective of this study was to investigate the optimal BP target and the effect of confounding factors on the clinical outcomes in patients undergoing prevalent dialysis.

## Methods

### Study subjects

**Figure 1:**
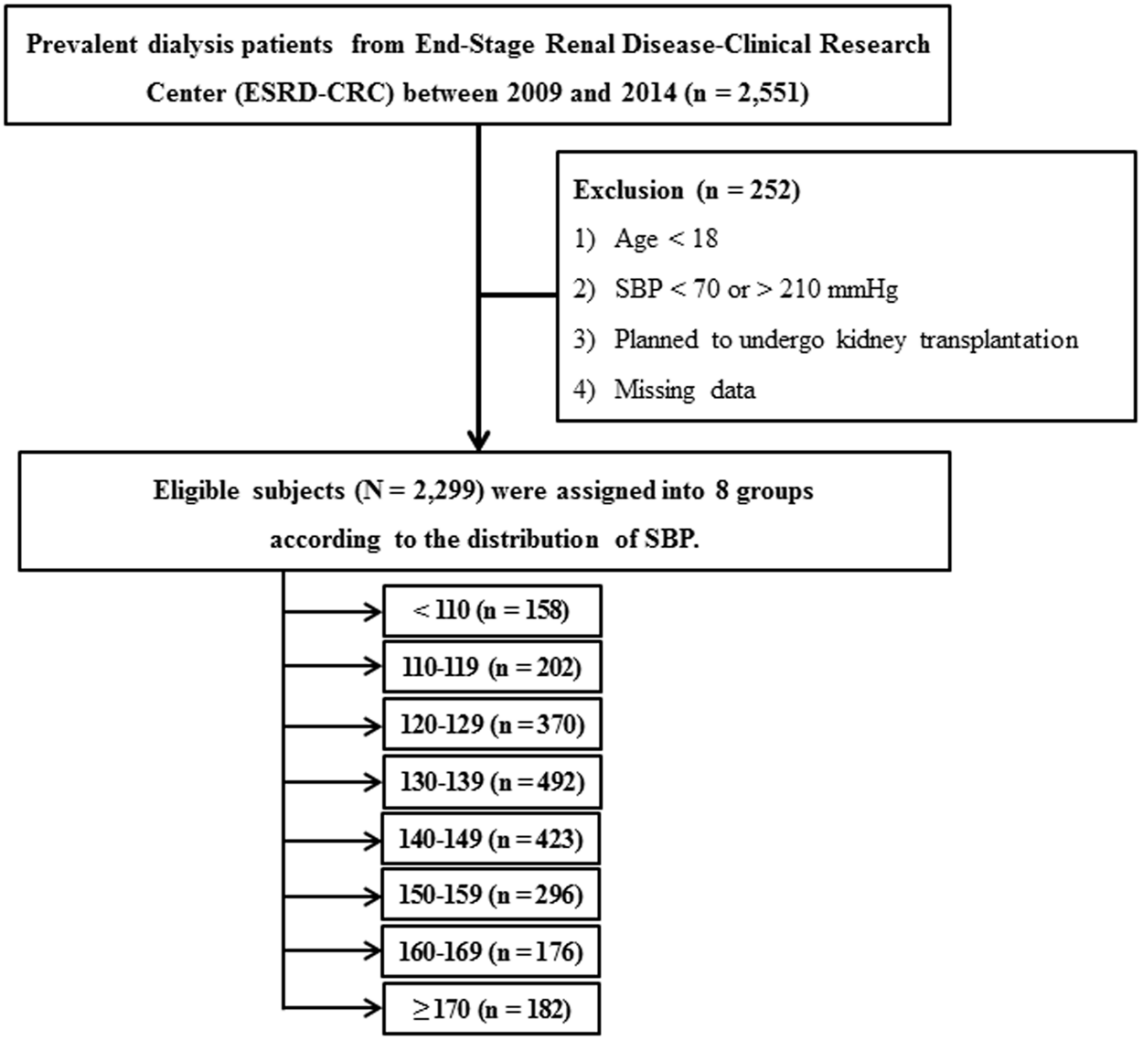
Flow diagram of the study cohort.

Data were retrieved from the Clinical Research Center for End-Stage Renal Disease, to which patients undergoing prevalent dialysis were prospectively enrolled from 2009 to 2014 in South Korea (ClinicalTrials.gov; NCT 00931970). This investigation was a nationwide, multicenter, web-based, prospective cohort study of patients with end-stage renal disease (ESRD) and was designed to improve the survival and quality of life of patients and create effective treatment guidelines^20^. Thirty-one hospitals participated in this cohort study. A total of 2,551 patients on prevalent dialysis (both HD and PD) were screened. A flow diagram depicting the process of participant selection is shown in Fig. 1. We excluded 252 patients who met the following criteria: 1) age <18 years, 2) SBP <70 or > 210 mmHg, 3) scheduled to undergo kidney transplantation, and 4) with missing data during follow-up. Finally, a total of 2,299 patients were included in the primary analysis. Eligible patients were assigned into eight groups according to the distribution of SBP (**<**110, 110–119, 120–129, 130–139, 140–149, 150–159, 160–169, and ≥170 mmHg). All included patients provided written informed consent, and the study was approved by the institutional review board of each center [The Catholic University of Korea, Bucheon St. Mary’s Hospital; The Catholic University of Korea, Incheon St. Mary’s Hospital; The Catholic University of Korea, Seoul St. Mary’s Hospital; The Catholic University of Korea, St. Mary’s Hospital; The Catholic University of Korea, St. Vincent’s Hospital; The Catholic University of Korea, Uijeongbu St. Mary’s Hospital; Cheju Halla General Hospital; Chonbuk National University Hospital; Chonnam National University Hospital; Chung-Ang University Medical Center; Chungbuk National University Hospital; Chungnam National University Hospital; Dong-A University Medical Center; Ehwa Womens University Medical Center; Fatima Hospital, Daegu; Gachon University Gil Medical Center; Inje University Pusan Paik Hospital; Kyungpook National University Hospital; Kwandong University College of Medicine, Myongji Hospital; National Health Insurance Corporation Ilsan Hospital; National Medical Center; Pusan National University Hospital; Samsung Medical Center, Seoul; Seoul Metropolitan Government, Seoul National University, Boramae Medical Center; Seoul National University Hospital; Seoul National University, Bundang Hospital; Yeungnam University Medical Center; Yonsei University, Severance Hospital; Yonsei University, Gangnam Severance Hospital; Ulsan University Hospital; Wonju Christian Hospital (in alphabetical order)]. All of the investigators conducted this study in accordance with the guidelines of the 2008 Declaration of Helsinki.

### Demographic and clinical parameters

Baseline information, including demographic and clinical data, was recorded at enrollment; other clinical and biochemical variables were obtained every 6 or 12 months. Demographic and clinical data included age, sex, height, body weight, comorbidities, dialysis information, and medication. During the baseline study visit, the classes and numbers of AHAs being taken were also investigated. Comorbidities included a history of DM, cardiovascular disease (CVD), and stoke. CVDs were defined as composites of coronary artery disease (CAD), myocardial infarction, and congestive heart failure. Predialysis SBP was measured by trained health-care professionals, with the patient in a sitting position. Predialysis venous blood samples were collected to establish the biochemical data, including hemoglobin, serum albumin, total cholesterol, low-density lipoprotein cholesterol (LDL-C), HbA1c, sodium, bicarbonate, ferritin, and high-sensitivity C-reactive protein. For calculating Kt/V in patients on HD, predialysis and postdialysis venous blood samples were obtained for the assessment of serum urea nitrogen. The 24 hr drained dialysates and urine were collected to calculate the Kt/V in patients undergoing PD. In patients on HD, IDWG was measured by using the predialysis and postdialysis weights.

### Study outcome

The primary outcome was all-cause mortality. Patient outcomes were assessed through December 31, 2014. During follow-up, patients were censored from the study at the time of death, kidney transplantation, or voluntary withdrawal.

### Statistical analysis

All statistical analyses were performed by using SPSS for Windows version 23.0 (SPSS Inc., Chicago, IL, USA) and SAS version 9.2 (SAS Inc., Cary, NC, USA). Continuous variables are expressed as mean ± standard deviation, whereas categorical variables are expressed as absolute numbers with percentages. Each variable was tested for normality before statistical analysis. Groups were compared by using analysis of variance or Student’s *t*-test for continuous variables, and the chi-square test or Fisher’s exact test for categorical variables. The Kolmogorov–Smirnov test was performed to determine the normality of the distribution of parameters. Data that did not show a normal distribution were presented as median and interquartile range. For these data, the Mann–Whitney U-test or Kruskal–Wallis test was used for multiple comparisons. Cox proportional hazards models were used to calculate the hazard ratios (HRs) for the association between SBP and mortality. Adjustments for all potential risk factors, including age, sex, comorbidities, dialysis vintage and type, numbers of AHAs, serum albumin, and LDL-C were done. For further analysis, patients were divided into subgroups according to age, the presence of DM or CAD, use of AHAs, dialysis type, dialysis vintage and the degree of IDWG (only for patients undergoing HD), and the risk for mortality in association with SBP was investigated by using Cox proportional hazard analysis. *P* values < 0.05 were considered statistically significant.

## Results

### Baseline characteristics of study subjects

The baseline characteristics of the study subjects are shown in Table 1. Their mean age was 62.3 ± 13.0 years, and 1306 (56.8%) patients were men. The mean value of predialysis SBP was 137.4 ± 20.7 mmHg. The patients were categorized into eight groups according to the distribution of SBP (Fig. 1). The mean value of SBP was 99.2, 119.7, 123.4, 132.6, 142.3, 152.1, 162.4, and 179.2 mmHg, respectively. There were no significant differences in age, dialysis vintage, and dialysis adequacy among the SBP groups. However, patients with higher SBP were taking more numbers of AHAs and showed a higher prevalence of DM. The baseline laboratory data revealed several differences; however, there were no significant correlative results across the eight groups.

**Table 1:** Baseline Characteristics of Patients Undergoing Prevalent Dialysis.

	Total (n = 2,299)	Distribution of SBP (mmHg)								
		<110 (n = 158)	110–119 (N = 202)	120–129 (N = 370)	130–139 (n = 492)	140–149 (n = 423)	150–159 (n = 296)	160–169 (n = 176)	≥170 (n = 182)	P
Age (years)	62.3 ± 13.0	61.9 ± 13.2	62.8 ± 13.3	61.6 ± 13.2	61.9 ± 12.8	63.0 ± 13.1	62.3 ± 12.9	62.8 ± 12.8	62.0 ± 13.0	0.91
Male (%)	1,306 (56.8)	75 (47.5)	105 (52.0)	204 (55.1)	288 (58.5)	254 (60.0)	173 (58.4)	104 (59.1)	103 (56.6)	0.37
Body mass index (kg/m^2^)	22.8 ± 3.3	22.2 ± 3.1	22.8 ± 3.4	22.7 ± 3.3	23.1 ± 3.4	23.0 ± 3.3	22.6 ± 33.4	22.8 ± 3.4	22.4 ± 3.2	0.07
Hemodialysis (%)	1,375 (59.9)	67 (42.4)	102 (50.7)	203 (54.9)	274 (56.0)	268 (63.4)	205 (69.5)	129 (73.3)	127 (69.8)	<0.001
Dialysis vintage (years)	8.7 ± 4.5	9.6 ± 5.2	8.2 ± 4.3	8.9 ± 4.8	8.6 ± 4.5	8.5 ± 4.5	9.0 ± 4.5	8.6 ± 4.4	8.7 ± 3.7	0.12
**Kt/V**										
Hemodialysis	1.4 ± 0.5	1.5 ± 0.6	1.5 ± 0.4	1.4 ± 0.5	1.4 ± 0.5	1.4 ± 0.5	1.5 ± 0.7	1.4 ± 0.4	1.4 ± 0.3	0.54
Peritoneal Dialysis	1.5 ± 0.5	1.5 ± 0.6	1.8 ± 0.2	1.4 ± 0.6	1.5 ± 0.6	1.5 ± 0.9	1.4 ± 0.4	1.5 ± 0.5	1.5 ± 0.4	0.05
Number of AHAs	1.9 ± 1.4	1.3 ± 1.4	1.6 ± 1.4	1.8 ± 1.5	1.9 ± 1.4	2.1 ± 1.4	2.2 ± 1.4	2.3 ± 1.3	2.4 ± 1.3	<0.001
**Classes of AHAs**										
ACEi/ARBs	1,271 (55.3)	65 (41.1)	106 (52.5)	189 (51.1)	258 (52.4)	237 (56.0)	179 (60.5)	115 (65.3)	122 (67.0)	<0.001
CCBs	1,167 (50.8)	45 (28.5)	81 (40.1)	164 (44.3)	247 (50.2)	231 (54.6)	177 (59.8)	113 (64.2)	109 (59.9)	<0.001
BBs	1,085 (47.2)	45 (28.5)	76 (37.6)	159 (43.0)	220 (44.7)	207 (48.9)	162 (54.7)	102 (58.0)	114 (62.6)	<0.001
alpha-blockers	246 (10.7)	6 (3.8)	12 (5.9)	40 (10.8)	55 (11.2)	53 (12.5)	39 (13.2)	18 (10.2)	23 (12.6)	0.02
**Comorbidities**										
DM (%)	730 (31.8)	48 (30.4)	49 (24.3)	94 (25.4)	150 (30.5)	152 (35.9)	87 (29.4)	73 (41.5)	77 (42.3)	0.001
CVD (%)	336 (14.6)	32 (20.3)	29 914.4)	60 (16.2)	70 (14.2)	54 (12.8)	40 (13.5)	29 (16.5)	22 (12.1)	0.36
Stroke (%)	30 (1.3)	4 (2.5)	2 (1.0)	3 (0.8)	10 (2.0)	5 (1.2)	2 (0.7)	3 (1.7)	1 (0.5)	0.46
**Laboratory data**										
Hemoglobin (g/dL)	10.7 ± 3.9	10.8 ± 1.6	10.6 ± 1.2	10.8 ± 1.3	10.6 ± 1.3	11.0 ± 5.6	11.0 ± 5.8	10.3 ± 1.2	10.7 ± 7.1	0.58
Albumin (g/dL)	33.9 ± 1.4	4.0 ± 2.7	3.8 ± 0.4	3.8 ± 0.4	3.9 ± 1.9	3.9 ± 1.7	3.8 ± 0.4	3.8 ± 0.4	3.7 ± 0.5	0.81
Total cholesterol (mg/dL)	162.4 ± 39.6	162.2 ± 39.8	166.7 ± 39.6	165.5 ± 40.1	166.3 ± 41.7	161.3 ± 38.6	159.0 ± 38.3	154.7 ± 35.7	156.9 ± 38.5	0.003
LDL-C (mg/dL)	92.0 ± 32.8	91.6 ± 29.5	96.5 ± 35.8	93.1 ± 32.0	96.0 ± 35.8	92.5 ± 33.2	87.4 ± 31.6	84.5 ± 27.9	90.1 ± 29.9	0.003
HbA1c (%)	6.5 ± 1.7	6.3 ± 1.6	6.2 ± 1.2	6.2 ± 1.4	6.4 ± 2.0	6.6 ± 1.7	6.6 ± 1.9	6.6 ± 1.6	6.6 ± 1.8	0.19
Sodium (mmol/L)	137.7 ± 6.0	137.6 ± 4.1	137.2 ± 10.2	138.3 ± 3.2	138.1 ± 3.5	137.7 ± 3.8	137.3 ± 8.6	136.8 ± 3.8	137.0 ± 10.2	0.03
Bicarbonate (mmol/L)	23.7 ± 8.6	24.2 ± 3.8	24.8 ± 11.2	24.1 ± 15.4	24.1 ± 4.0	23.4 ± 5.6	23.5 ± 9.5	22.0 ± 3.5	23.1 ± 4.4	0.28
Ferritin (ng/mL)^a^	203.2 [101.0–382.3]	224.7 [79.7–405.3]	205.2 [109.6–367.7]	199.8 [94.0–373.0]	192.9 [96.5–391.7]	200.6 [98.8–372.1]	179.3 [101.8–332.2]	213.6 [115.0–370.3]	246.6 [129.1–429.5]	0.38
hs-CRP ^a^(mg/dL)	0.13 [0.02–0.55]	0.14 [0.04–0.51]	0.11 [0.01–0.59]	0.12 [0.02–0.39]	0.13 [0.02–0.68]	0.12 [0.02–0.52]	0.17 [0.01–0.58]	0.17 [0.04–0.72]	0.12 [0.01–0.61]	0.66

Note: ^a^Kruskal-Wallis test.

Abbreviations: AHA, antihypertensive agent; ACEi, angiotensin converting enzyme inhibitor; ARB, angiotensin II receptor blocker; CCB, calcium channel blocker; BB, beta-blocker; DM, diabetes mellitus; CHF, congestive heart failure; CVD, cardiovascular disease; LDL-C, low-density lipoprotein-cholesterol; hs-CRP, high-sensitivity C-reactive protein.

### BP and all-cause mortality

**Figure 2:**
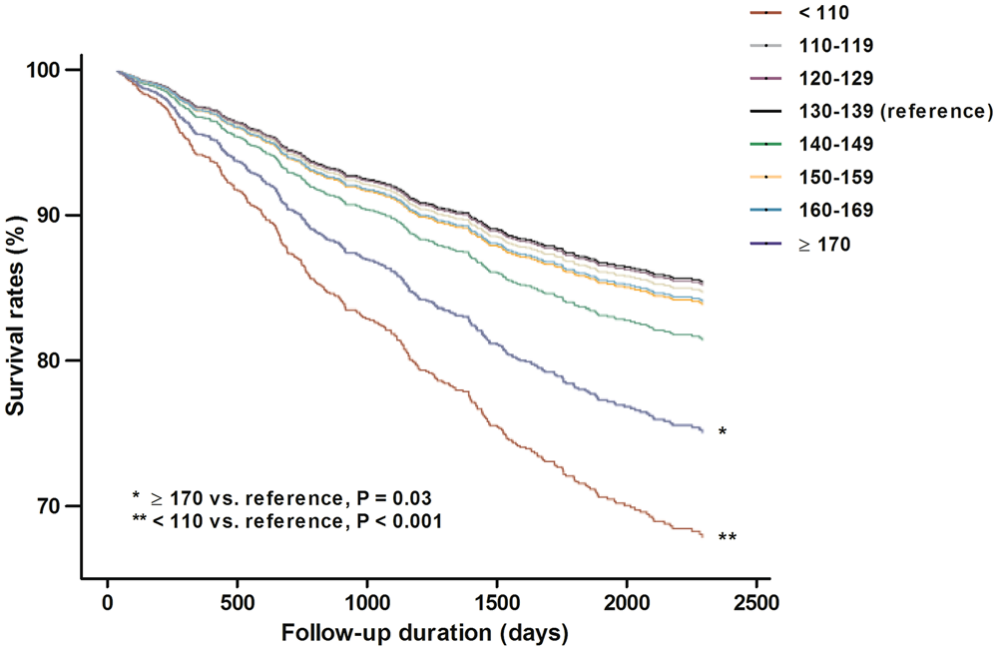
Kaplan-Meier analysis for all-cause mortality among the eight systolic blood pressure groups.

**Figure 3:**
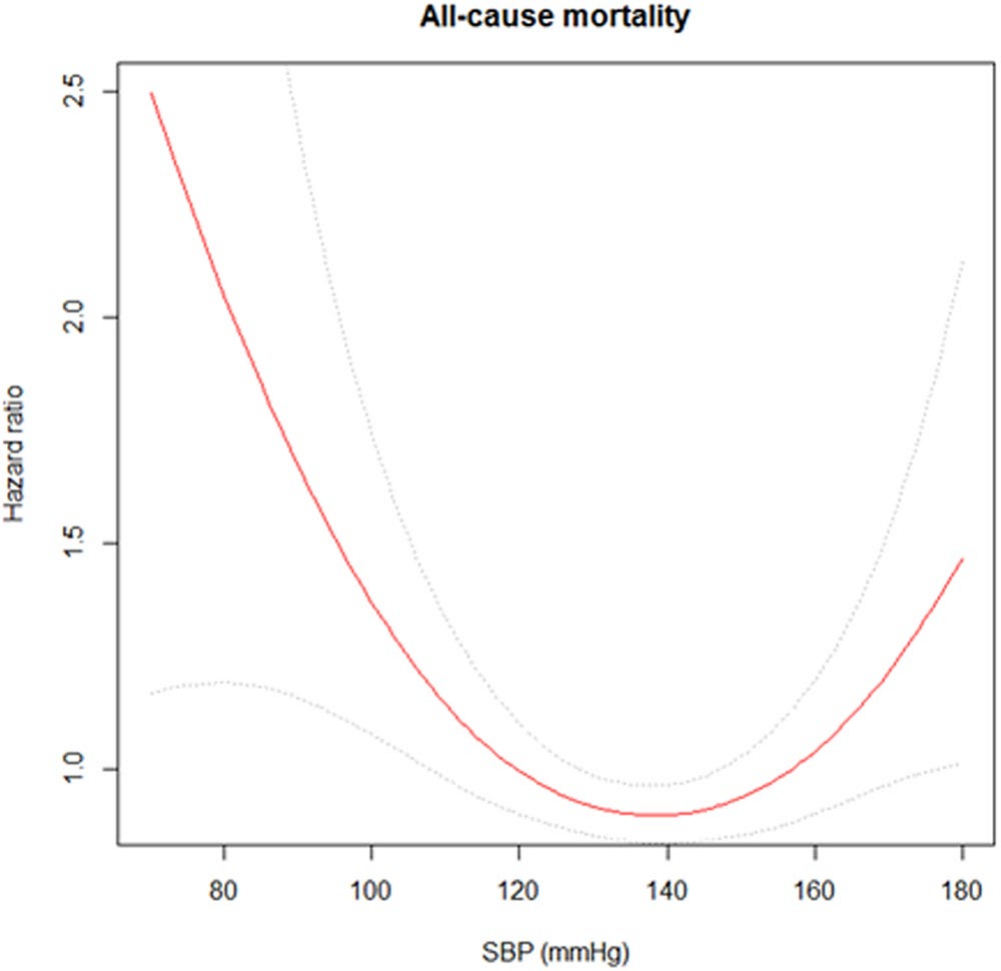
Cubic spine curve for all-cause mortality with predialysis systolic blood pressure.

During the median follow-up of 4.5 years, 45 (28.5%), 27 (13.4), 48 (13.0), 80 (16.3), 60 (14.2), 42 (14.2), 26 (14.8), and 40 (22.1) patients reached the primary end point in the each SBP group, respectively. Kaplan–Meier survival analysis was done to investigate the association between all-cause mortality and SBP. The results showed that the groups with SBP <110 and ≥170 mmHg were significantly associated with a higher risk of mortality compared to SBP 130–139 mmHg as a reference group (Fig. 2). Univariable Cox analysis was done to identify the risk factors for all-cause mortality. Age, sex, number of AHAs, dialysis type and vintage, history of DM or CVD, serum albumin, and LDL-C levels were associated with all-cause mortality (data not shown). Subsequently, multivariable Cox analysis with adjustment for confounding factors was done. The results still showed that the <110 and ≥170 mmHg SBP groups were associated with a higher risk of all-cause mortality (HR, 1.68; 95% confidence interval (CI), 1.14–2.49; P = 0.01 and HR, 1.63; 95% CI, 1.10–2.42; P = 0.02 in groups with SBP <110 and ≥170 mmHg, respectively) (Table 2). SBP groups with 110–119, 120–129, 140–149, and 150–159 mmHg showed decreased risk of all-cause mortality than SBP group with 130–139 mmHg, however there were no statistical significances. Finally, the cubic spline with HR plot revealed a U-shaped curve relation between SBP and all-cause mortality (Fig. 3).

**Table 2:** Multivariate Cox Analysis for All-cause Mortality among the Five Patient Groups.

	Crude^a^		Model 1^b^		Model 2^c^	
	HR (95% CI)	P	HR (95% CI)	P	HR (95% CI)	P
**Distribution of SBP (mmHg)**						
<110	1.85 (1.28–2.66)	**<**0.001	1.93 (1.34–2.79)	**<**0.001	1.68 (1.14–2.49)	0.01
110–119	0.81 (0.52–1.25)	0.35	0.75 (0.48–1.16)	0.19	0.76 (0.48–1.19)	0.23
120–129	0.79 (0.55–1.13)	0.20	0.78 (0.55–1.12)	0.18	0.78 (0.54–1.14)	0.20
130–139	Reference		Reference		Reference	
140–149	0.88 (0.63–1.23)	0.45	0.81 (0.58–1.13)	0.21	0.85 (0.60–1.21)	0.36
150–159	0.86 (0.59–1.25)	0.42	0.82 (0.57–1.20)	0.31	0.94 (0.64–1.39)	0.77
160–169	0.91 (0.58–1.41)	0.67	0.83 (0.53–1.29)	0.41	1.02 (0.65–1.62)	0.92
≥170	1.41 (1.01–2.05)	0.03	1.37 (1.02–2.01)	0.02	1.63 (1.10–2.42)	0.02

Note: ^a^Unadjusted model.

^b^Adjusted model for age, sex, history of DM, CVDs.

^c^Adjusted model for model 1 + dialysis vintage, dialysis type, number of AHAs, LDL-C, and serum albumin.

Abbreviations: HR, hazard ratio; CI, confidence interval; SBP, systolic blood pressure; AHA, antihypertensive agents; LDL-C, low-density lipoprotein cholesterol.

### Subgroup analysis

**Figure 4:**
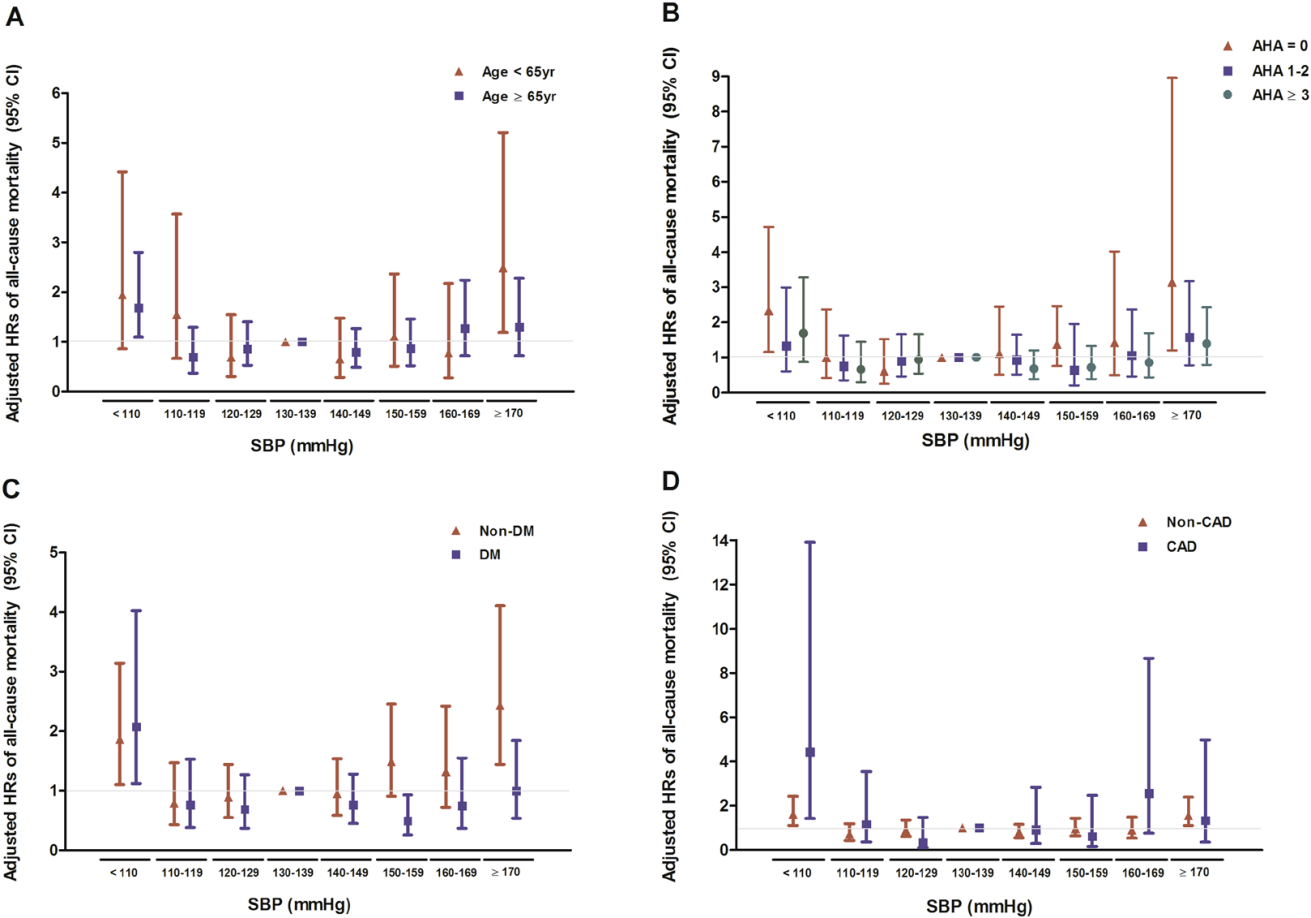
Adjusted hazard ratios (95% confidence intervals) of all-cause mortality with predialysis SBP categories in subgroups; (**A**) age (**<**65 years vs. ≥65 years), (**B**) number of AHAs (0 vs. 1–2 vs. ≥3), (**C**) history of DM (DM vs. non-DM), and (**D**) history of CAD (CAD vs. no CAD). Model is adjusted for age, sex, history of DM or CAD, dialysis vintage, number of AHAs, LDL-C, and serum albumin.

**Figure 5:**
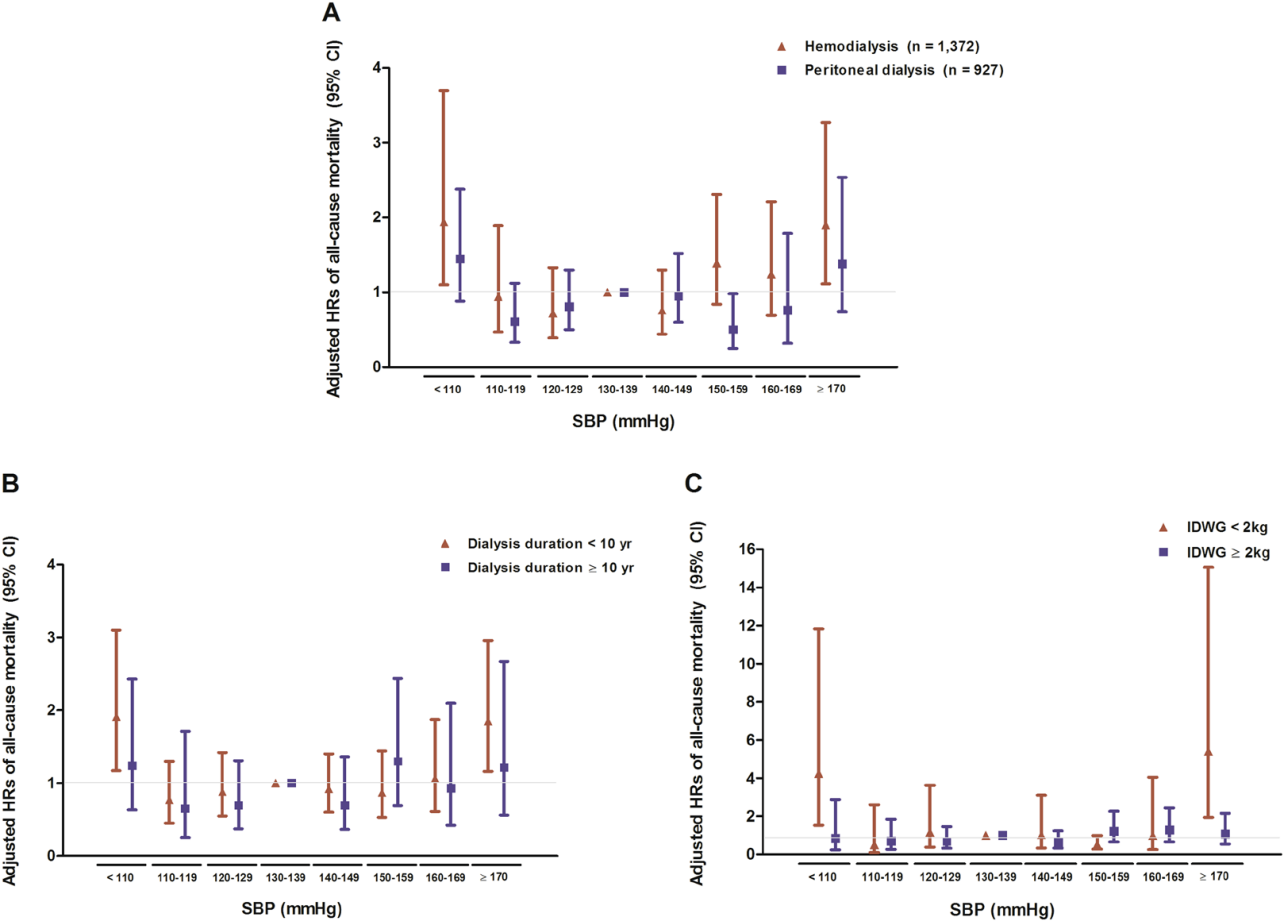
Adjusted hazard ratios (95% confidence intervals) of all-cause mortality with predialysis SBP categories in subgroups; (**A**) type of dialysis (HD vs. PD), (**B**) dialysis vintage (**<**10 years vs. ≥10 years), and (**C**) IDWG (**<**2 kg vs. ≥2 kg). Both (**B**,**C**) are evaluated only in HD patients (n = 1,379). Model is adjusted for age, sex, history of DM or CAD, dialysis vintage, number of AHAs, LDL-C, and serum albumin.

Subgroup analysis was done to investigate whether the relationship between SBP and all-cause mortality would be influenced by several clinical factors. First, patients were divided into subgroups according to age, presence of DM or CAD, and use of AHAs. Second, patients were divided by the type of dialysis (HD or PD). Finally, for HD patients, subjects were divided into two groups according to dialysis vintage and IDWG. Figure 4 shows the results of subgroup analysis among all study subjects. Figure 5 shows the results of subgroup analysis for HD vs. PD, and according to dialysis characters among HD patients. The precise descriptions of the results are provided below.

### Age, presence of diabetes or coronary artery disease, and use of AHAs

Subjects were first divided into subgroups according to age (<65 or ≥65 years), history of DM or CAD, and use of AHAs. In subgroups with no history of DM or CAD, as well as lack of AHA usage, SBP and all-cause mortality showed similar U-shape relationships to the ones shown in primary results described above. However, in subgroups with age ≥ 65 years and having DM or CAD, only SBP <110 mmHg was significantly related to higher risks for mortality. In subgroups with age <65 years, only highest SBP was associated with increased risk for mortality (Fig. 4).

### Type of dialysis (HD vs. PD)

Among patients undergoing HD, adjusted HR was higher in SBP <110 and ≥170 mmHg groups (HR, 1.94; 95% CI, 1.02–3.71; P = 0.04 and HR, 1.90; 95% CI, 1.11–3.27, respectively; P = 0.02). In patients undergoing PD, a similar trend was observed (i.e., the lowest and highest SBP groups were related to a higher risk of mortality); however, there was no statistical significance (Fig. 5A).

### Dialysis vintage and IDWG in patients undergoing HD

Among patients undergoing HD, subjects were divided into two groups according to dialysis vintage. Interestingly, SBP <110 and ≥170 mmHg were significantly associated with an increased risk of all-cause mortality in subjects with <10 years of dialysis vintage. Although the group with SBP <110 and ≥170 mmHg showed a higher risk of mortality, no statistical significance was found in patients with a long dialysis vintage (Fig. 5B). Among patients undergoing HD whose IDWG was <2 kg, the results were closely parallel to previous results. Patients in SBP <110 and 170 mmHg subgroups were significantly associated with an increased risk of all-cause mortality. However, in patients with greater IDWG, no significant relationship between SBP and mortality was observed (Fig. 5C).

## Discussion

In this study, we found a U-shaped relation between predialysis SBP and all-cause mortality. In particular, patients in subgroups with no history of DM or CAD and no use of AHAs, who are relatively healthy compared to those in opposite groups, showed similar U-shaped relationship in terms of SBP and the primary outcome. However, in subgroups for those with over 65 years of age, history of DM or CAD, only lower SBP (**<**110 mmHg) seemed to significantly increase risk for mortality. In regards to dialysis characteristics, HD patients showed similar relation with primary results, while PD patients showed no relevant results between SBP and mortality. Specifically, in HD patients with shorter dialysis vintage and IDWG <2 kg, SBP and mortality showed U-shaped relationship; meanwhile, HD patients with longer dialysis vintage and IDWG ≥ 2 kg did not.

The optimal BP target is long-lasting and unsettled issue not only in general population but also in patients with advanced chronic kidney disease. Recently, intensive BP control is suggested to be beneficial for clinical outcomes in general population^4^. 2017 American College of Cardiology/American Heart Association (ACC/AHA) guidelines lower the SBP criteria of hypertension that SBP higher than 130 mmHg to be hypertension^21^. In contrast, BP target is far more complex and difficult to determine in dialysis population. Mostly, an inverse (U-shaped) relation between predialysis SBP and mortality rates has been reported in the dialysis population^6,8,22^. To explain the U-shaped relation, previous studies proposed several contributing factors such as underlying heart failure and low cardiac output status, survival bias, competitive risk factors, or neurohormonal state unique to patients undergoing HD^23–25^. Hannedouche *et al*.^8^ explained the mechanism by which lower BP is related to higher mortality. According to the authors, the inability to raise BP in response to the accumulation of fluid between HD sessions may represent a loss of compensatory and physiologic response of a patient to be relatively healthy^26^. Moreover, patients with lower baseline BP are more prone to intradialytic hypotension and may develop progressive organ hypoperfusion during intradialytic ultrafiltration^13,27^. Thus, a lower BP may be a sign of comorbid conditions not entirely represented by other scores^28^.

Some of study groups suggest different BP targets from general population should be determined in high risk patients such as those with old age or more comorbidities. Particularly, observational studies have found U- or J-shaped relationships of BP with mortality in older patients^29^. Moreover, increasing evidences are reported that patients with diabetes or coronary artery disease have higher risk of mortality with lowering SBP less than 130 mmHg, showing the discrepancy in BP target from general population^30,31^. Lower SBP in these patients was rather associated with extensive hypotension^30^. Thus, treatment implications for BP control among high risk patients remain unsolved and it can be inferred that different BP targets should be determined differentially with consideration for patient’s disease characteristics. Therefore, considering that dialysis population is a high-risk group who are relatively old and having great numbers of comorbidities, BP target may differ from general population and cannot be determined uniformly. Thus, our study suggests that the lowest (**<**110 mmHg) and highest (≥170 mmHg) SBPs are associated with risk for mortality, whereas SBP ranging 110 to 169 mmHg might have decreased risk without significances. Nevertheless, BP target should be individualized with consideration for patient’s characteristics.

For the above mentioned reasons, this study has strengths in that we further conducted subgroup analysis with each of the clinical factors that are known to substantially affect the association between BP and mortality. We divided the subjects according to age <65 or ≥65 years. The relation of SBP to mortality was not uniform across the subgroups, suggesting that the optimal BP target may differ according to age. Elderly patients might have stiffer and noncompliant vessels, and are frequently prone to occlusive arterial disease, which result in significant reductions in organ perfusion during dialysis^6^. Our study also demonstrated that elderly patients (>65 years old) with low SBP, but not those with high SBP, were significantly associated with an increased risk of mortality. In respect to DM, patients without DM revealed similar results to the previous analysis (i.e., the risk of mortality was dually associated with increased or decreased in SBP). However, in patients with DM, only lower SBP was significantly associated with an increased risk of mortality. A previous study performed by Ishida *et al*.^32^ reported that HD causes severe orthostatic hypotension which lead to reduction in cerebral blood flow velocity in diabetic patients. It can be explained that vulnerability of ischemic damage resulting from low SBP increase the risk of adverse outcome among DM patients. Moreover, lowering the SBP is only beneficial in patients without CAD, and not in patients with CAD. There are still having conflicts about BP effects on cardiovascular outcomes. However, some of previous studies suggested that a higher BP could have a protective role in improving cardiac perfusion or overcoming the resistance of stiff arteries in patients with CAD^5,33–35^. This is in line with present study, that patients with CAD have no benefits of lowering the SBP. In regard of AHAs, Agarwal *et al*.^14^ reported that use of AHAs is not correctly related to BP. They also suggested that adequate management of the volume status is closely related to BP control, and excessive use of AHAs may interfere with the achievement of DW and paradoxically increase the predialysis BP. The present study also revealed a significant U-shape association between SBP and mortality rate only in patients with no use of AHAs, whereas this relationship was not constant in patients who were using AHAs. Taken together, the optimal BP target should be stratified carefully according to age, the presence of DM or CAD, and use of AHAs.

We also investigated the impact of SBP on the patient outcomes according to the several dialysis characters including dialysis type, vintage, and IDWG. Studies investigating the association between BP and mortality are scarcer in patients on PD than in patients on HD. Udayaraj *et al*.^19^ reported that a greater SBP in the first year after the start of PD was significantly associated with decreased mortality. Meanwhile, other studies^18,36–38^ showed a significant association between lower SBP and higher mortality in patients on PD. This study showed similar trend of BP target in patients on PD compared to patients on HD. However there were no statistical significant results among patients on PD. Further study is needed to identify optimal target of SBP in patients on PD. Dialysis vintage also affects the target SBP among HD patients. The results of our study showed that SBP <110 or ≥170 mmHg significantly increased the risk of mortality in patients undergoing HD with a dialysis vintage of <10 years. However, patients with a longer dialysis vintage showed significantly increased mortality only with SBP <110 mmHg. Stidley *et al*.^39^ investigated patients undergoing HD to investigate the effect of dialysis vintage on BP management. They concluded that low SBP was associated with increased mortality within 2 years; conversely, high SBP was associated with increased mortality among patients who survived for >3 years. We can postulate that several factors affecting BP, including response to the renin–angiotensin–aldosterone system, vascular calcification, and arterial stiffness are changed, which result in such discrepancies in the association of BP and mortality between patients with short and long dialysis vintage. Further studies should be performed to identify the possible exact mechanism for this issue. Maintaining DW or chronic volume overload according to adequate ultrafiltration is the main reason for uncontrolled hypertension in patients undergoing dialysis^40^. In the present study, patients undergoing HD with poorly controlled DW, an L-shaped, not a U-shaped, association between SBP and mortality was observed. The large IDWG indicates large volume of ultrafiltration during dialysis, which easily result in hypovolemia and intradialytic hypotension^41^. In addition, the volume overload status gives more pressure or volume stress on the heart and the low BP might make more intolerable to stand for these. Accordingly, theses mechanisms might have an effect on low SBP to increase risk of mortality.

This study has several limitations. First, our data set did not include various measurements of BP including postdialysis, interdialytic, and ambulatory BP. Several studies have suggested that ambulatory BP is reliable in predicting the clinical outcomes in patients on dialysis despite its practical inconvenience^42,43^. On the other hand, predialysis SBP has the merit of being easily applicable. In this regard, it might be more practical to use the easily assessable BP. Second, we did not analyze BP as a time-varying variable, because only the baseline BP measurement was available from our data set. In addition, several other contributing factors to the BP value, such as dietary sodium intake, were not included as confounding factors. Further studies with time-varying covariates of BP and other confounding factors might have more statistical power and produce more relevant clinical results. Finally, the disease period of hypertension was not included in the analysis. Since long-tern hypertension can be a strong risk factor for target organ damage and leads to adverse clinical outcomes in dialysis population^44^, lack of data regarding duration of hypertension in this study made limitation and further investigation is warranted.

In conclusion, the survival rates were significantly worse in the highest and lowest SBP groups among patients undergoing chronic dialysis. SBP at 110–169 mmHg showed no differences on the risk for all-cause mortality. Furthermore, the optimal SBP target differed among each subgroup stratified according to age, history of DM or CAD, use of AHAs, dialysis type and vintage, and the degree of IDWG among patients on prevalent dialysis. Maintaining SBP at ranges of 110–169 mmHg is applicable for dialysis patients with young, having no comorbidities, and taking no AHAs as well as undergoing HD with shorter dialysis vintage and less IDWG. However, in patients with older and having comorbidities, maintaining SBP at >110 mmHg is only beneficial, whereas lowering the SBP to <170 mmHg is not. The optimal target BP should be individually applied according to patient’s condition.

## Data Availability

Due to legal restrictions, data are made available upon request from the CRC (clinical research center) for ESRD study. Patients only approved of opening their data to the research center and data are available under the authority of the management committee of CRC for ESRD. Please contact Yong-Lim Kim, MD, PhD. Kyungpook National University School of Medicine. Tel: + 82-53-200-5553, E-mail: ylkim@knu.ac.kr.
